# CD8+ lymphocytes/ tumour-budding index: an independent prognostic factor representing a ‘pro-/anti-tumour’ approach to tumour host interaction in colorectal cancer

**DOI:** 10.1038/sj.bjc.6605318

**Published:** 2009-09-15

**Authors:** A Lugli, E Karamitopoulou, I Panayiotides, P Karakitsos, G Rallis, G Peros, G Iezzi, G Spagnoli, M Bihl, L Terracciano, I Zlobec

**Affiliations:** 1 Institute of Pathology, University of Basel, Schönbeinstrasse 40, Basel 4031, Switzerland; 2 Second Department of Pathology, Attikon University Hospital, Rimini 1, Haidari, Athens 12464, Greece; 3 Department of Diagnostic Cytopathology, Attikon University Hospital, Rimini 1, Haidari, Athens 12464, Greece; 4 Fourth Department of Surgery, Attikon University Hospital, Rimini 1, Haidari, Athens 12464, Greece; 5 Institute for Surgical Research and Hospital Management, University of Basel, Hebelstrasse 20, Basel 4031, Switzerland

**Keywords:** tumour budding, CD8+ lymphocytes, colorectal cancer, prognosis

## Abstract

**Background:**

The tumour-host interaction at the invasive front of colorectal cancer, including the epithelial–mesenchymal transition and its hallmark ‘tumour budding’, is an important area of investigation in terms of prognosis. The aim of this study was to determine the prognostic impact of a ‘pro-/anti-tumour’ approach defined by an established ‘pro-tumour’ (tumour budding) and host-related ‘anti-tumour’ factor of the adaptive immunological microenvironment (CD8+ lymphocytes).

**Methods:**

Double immunostaining for CK22/CD8 on whole tissue sections (*n*=279; Cohort 1) and immunohistochemistry for CD8+ using tissue microarrays (*n*=191; Cohort 2) was carried out. Tumour buds, CD8+ and CD8+ T-lymphocytes : tumour buds indices were evaluated per high-power field.

**Results:**

In Cohort 1, a low-CD8+/ buds index was associated with lymph node metastasis (*P*<0.001), vascular invasion (*P*=0.009), worse survival in univariate (*P*<0.001) and multivariable (*P*<0.001) analysis, and furthermore in lymph node-negative patients (*P*=0.002). In Cohort 2, the CD8+/ buds index was associated with T stage (*P*<0.001), N stage (*P*=0.041), vascular invasion (*P*=0.005) and survival in patients with TNM stage II (*P*=0.019), stage III (*P*=0.004), and adjuvantly untreated (*P*=0.009) and treated patients (*P*<0.001).

**Conclusion:**

The CD8+ lymphocyte : tumour-budding index is an independent prognostic factor in colorectal cancer and a promising approach for a future prognostic score for patients with this disease.

During the Napoleonic Wars (1796–1815), one of the most effective methods to attack in battle was the deployment of heavy cavalry, which the opponent tried to repel by organizing the infantry into squares.

A similar picture can be observed at the invasion front of colorectal cancer. On one hand, the tumour mass invades the pericolic fat tissue, detaching clusters or single tumour cells (tumour buds) reflecting tumour progression; whereas, on the other hand the host attempts to confront this situation by building an ‘anti-tumour’ cytotoxic inflammatory response. This ‘pro-/anti-tumour’ model is supported by a range of studies proposing either independent tumour-related prognostic factors (pro-tumour), such as tumour grade, tumour border configuration, medullary subtype, CEA level, microsatellite instability, loss of heterozygosity (LOH) 18q status, p53 levels, TGFB1 type II receptor levels, VEGF expression, proliferation rate and metalloproteinase expression (Compton, 2006) or anti-tumour factors, such as CD3+, CD4+, CD8+, CD20+ lymphocytes, Granzyme B, FoxP3+ regulatory T cells (Tregs), CD16+ cells, and mast and dendritic cells (Dadabayev *et al*, 2004; Phillips *et al*, 2004; Sato *et al*, 2005; Chaput *et al*, 2009; Salama *et al*, 2009).

Consequently, several groups focus on the invasive front of colorectal cancer using the term epithelial–mesenchymal transition (EMT), which characterises tumour invasion by de-differentiated colorectal carcinoma cells (Brabletz *et al*, 2005). EMT and MET, the reverse transition from a mesenchymal to an epithelial phenotype, are crucial steps not only in embryonic development but also in tumour progression (Spaderna *et al*, 2007).

A histomorphological hallmark of EMT is the phenomenon of ‘tumour budding’ (Prall, 2007), which according to the third edition of ‘Prognostic Factors in Cancer’ published by the UICC in 2006, is considered an additional prognostic factor in colorectal cancer (Compton, 2006). A tumour bud is typically defined as a single tumour cell or tumour cell cluster of up to five cells at the invasive tumour front (Prall, 2007). Indeed, tumour budding has been shown to be associated with lymph node positivity, poorly differentiated tumours, presence of vascular and lymphatic invasion, local tumour recurrence and distant metastasis (Ueno *et al*, 2004a,2004b; Nakamura *et al*, 2005; Kazama *et al*, 2006; Ishikawa *et al*, 2008; Wang *et al*, 2009). In particular, patients with stage III disease have been reported to demonstrate a 5-year disease-free survival (DFS) of 62.1% in the absence of tumour budding and only a 35.1% DFS with this feature (Choi *et al*, 2007). Moreover, the presence of tumour budding has repeatedly been linked to poor clinical outcome, underlined by the adverse effect on overall survival independent of TNM stage (Hase *et al*, 1993; Ueno *et al*, 2004b).

Over the last 20 years, investigations on tumour immunity and host defence in colorectal cancer demonstrate promising results for immunotherapy. In most colorectal cancers, lymphocytic infiltration is composed predominantly of either CD4+ or CD8+ T cells and both cell types appear to be significantly increased in tumour as compared with normal tissue (Ropponen *et al*, 1997; Naito *et al*, 1998; Chiba *et al*, 2004; Koch *et al*, 2006). Several studies have shown that tumour infiltrating lymphocytes (TILs) within the stroma and around the tumour along the invasive margin are significantly related to overall- and disease-specific survival in both univariate and multivariable analysis (Ali *et al*, 2004; Canna *et al*, 2005; Pages *et al*, 2005). Galon *et al* (2006) evaluated by gene-expression profiling and immunohistochemistry, the type, density and location (whether at the invasive margin or the tumour centre) of TILs in a large number of cases. They evaluated CD3, CD8, granzyme B and memory CD45RO T cells, demonstrating a significant independent and positive effect of TILs on both recurrence and survival.

In colorectal cancer, mismatch-repair status (microsatellite stable (MSS) and microsatellite instability-high (MSI-H)) seems to relate highly to the number of CD8+ lymphocytes. Compared with MSS tumours, MSI-H cancers are characterised by prolonged survival time, significantly more frequent peritumoural lymphocytic infiltration at the invasive front and by an inherent abundance of intra-epithelial TILs (Jass *et al*, 1998; Michael-Robinson *et al*, 2001; Greenson *et al*, 2003; Jenkins *et al*, 2007). Nevertheless, many studies analyzing colorectal cancer samples stratified by mismatch-repair status also report a positive effect of CD8+ lymphocytes in mismatch-repair proficient colorectal cancers (Baker *et al*, 2007).

Although this wide range of possible additional prognostic factors in colorectal cancer is currently being investigated, still missing is an approach to include parameters reflecting the tumour dynamics. Such an approach has already been taken for the prognostication of breast cancer, namely the Bloom–Richardson–Elston (BRE) score, which encompasses information on mitoses, tubule differentiation and nuclear pleomorphism, and is used as an important prognostic feature additional to TNM staging.

Therefore, the aim of this study was to investigate on a potential ‘pro-/anti-tumour’ model and to test the prognostic impact of a ratio defined by an established pro-tumour (tumour budding) and anti-tumour (CD8+ lymphocytes) factor. To this end, two independent colorectal cancer patient cohorts from different centres were investigated using two different approaches, namely whole tissue sections (*n*=300) and the tissue microarray technique (*n*=221).

## Materials and methods

### Cohort 1-Whole tissue sections

#### Sample size determination

To reach 85% power with an expected risk ratio of 1.8 between prognostic groupings and potential loss of patient samples in 10% of cases the appropriate sample size for this study was determined to be 255 cases. Owing to the availability of material this number was increased to 300 cases.

*Specimens*: Paraffin-embedded tissue blocks of 300 resection specimens of patients treated between 1987 and 1996 at the University Hospital of Basel were retrieved from the archives of the Institute of Pathology, University Hospital of Basel, as well as at the Institute of Clinical Pathology, Basel, Switzerland. These 300 cases were randomly selected from a larger previously described cohort of 938 colorectal cancer patients with full clinico-pathological information (Zlobec *et al*, 2008). The use of material for this study was approved by the local research ethics committee.

#### Double immunostaining for CD8 and CK22

**Figure 1:**
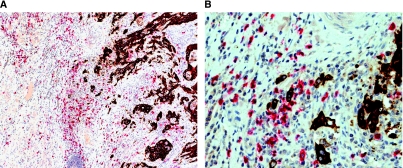
Double immunostaining for CD8 (red) and CK22 (brown): (**A**) Overview ( × 10) and (**B**) high power field ( × 40) of the invasive front of colorectal cancer showing CK22 positive tumour buds surrounded by CD8+ T-lymphocytes.

A double immunostaining procedure using anti-CD8 (for detection of CD8+ T-lymphocytes) and pan-cytokeratin (to facilitate visualization of tumour buds at the invasive front) was carried out on one representative slide cut at 4 *μ*m from paraffin-embedded tumour blocks of all 300 colorectal cancer patients included in this study (Figure 1). Double staining was carried out using the BOND-MAX Automated Immunohistochemistry Vision Biosystem (Leica Microsystems GmbH, Wetzlar, Germany) according to the following protocol. First, tissues were deparaffinised and pre-treated with the Epitope Retrieval Solution 2 (EDTA-buffer pH8.8) at 100°C for 20 min. After wash steps, peroxidase blocking was carried out for 10 min using the Bond Polymer Refine Detection Kit DC9800 (Leica Microsystems GmbH). Tissues were again washed, then incubated with primary antibody against CK22 (Biomeda, Foster City, CA, USA, pan-CK22) for 30 min. Subsequently, tissues were incubated with polymer for 15 min and then with DAB-Chromogen for 10 min (Bond Polymer AP Red Detection Kit DS9305, Leica Microsystems GmbH). CK22 positive cells were therefore coloured in brown. After washing, incubation was carried out with anti-CD8 (DakoCytomation, Glostrup, Denmark, clone CD8/144B) for 30 min followed by application of AEC-substrate for 10 min and counterstaining with haematoxylin for 2 min.

#### Evaluation of tumour buds and CD8+ T-lymphocytes

Tumour budding was defined as an isolated single cancer cell or a cluster of up to five cells at the invasive front of colorectal cancer (Prall, 2007). The tumour border was scanned at a × 100 magnification and the area of most intense budding was identified (Ueno *et al*, 2002). After selecting this field, the number of buds was counted using a 40 × objective lens to focus specifically on the presence of CD8+ T-cells most highly related to the microenvironment surrounding the tumour buds. Within this same field, all CD8+ lymphocytes were individually counted. In 10 cases, the abundance of CD8+ infiltrate led to cell counts exceeding 200 cells per field, and thus single cell counting was not feasible. These cases were assigned a CD8+ score of 200 cells. The ratio of CD8+ T-lymphocytes to the number of tumour buds (CD8+/ buds index) was obtained. In 13 cases when zero buds were identified, the count of CD8+ lymphocytes was not carried out.

#### Clinico-pathological characteristics

Of these 300 cases, 279 were evaluable for CD8 and CK22 protein expression, simultaneously. Hematoxylin and eosin (H&E) stained slides were reviewed and histomorphological data included histological subtype, pT stage, pN stage and tumour grade. The tumour border configuration and the presence of conspicuous peritumoural lymphocytic infiltration were defined according to Jass *et al* (1986). Clinical data were retrieved from patient records and included age at diagnosis, gender, tumour location and follow up. Clinical outcome of interest was disease-specific survival time, which was available for all 279 patients. Median follow-up time was 60 months. In all, 128 patients died of disease. Patient characteristics are listed in Table 1.

#### Microsatellite instability (MSI) status, KRAS and BRAF gene status

Genomic DNA was obtained from primary tumours using NucleoMag 96 Tissue Kit (Macherey Nagel, Oensingen, Switzerland) protocol and processed in the Xiril X-100 robot (Xiril, Hombrechtikon, Switzerland). Briefly, punched tissue was lysed in proteinase K. B-beads and MB2 buffer were added to the cleared lysate and shaken for 5 min at room temperature. The supernatant was removed and MB3 was added followed by shaking and supernatant removal. The genomic DNA was eluted with MB6 buffer. Genomic DNA was amplified by PCR using AmpliTaq Gold polymerase (Applied Biosystem, Foster City, CA, USA). *KRAS* (exon 2, codon 12 and 13) and *BRAF* (exon 15, codon 600) were amplified by a first and a nested PCR. Residual primers were removed using the EXOSAPit (Amersham, Otelfingen, Switzerland). Samples were then subjected to direct sequencing of single-stranded PCR products using the BigDye Terminator v1.1 cycle sequencing kit (Applied Biosystems) and the ABI Prism 3130 genetic analyser (Applied Biosystems). All products were sequenced bi-directionally. Analysis of MSI status was based on the multiplex amplification of the five microsatellites (BAT25, BAT26, D2S123, D5S346 and D17S250). An initial denaturation step at 95°C for 10 min was followed by 42 cycles at 95°C for 40 s, 54°C for 40 s and 72°C for 60 s. For the analysis, 1 *μ*l of the DNA weight marker ROX 500 (Applied Biosystem) was added and 10 *μ*l of deionised formamide in 3 *μ*l of the PCR amplified solution. DNA was denaturated by incubation for 2 min at 95°C. The POP-7 polymer solution (Applied Biosystem) was used for the electrophoresis on the ABI Prism 3130 genetic analyser (Applied Biosystems). MSS and MSI-low (MSI-L) status were defined as instability at zero and one markers, respectively. MSI-H was characterised by the presence of instability in two or more markers (Umar *et al*, 2004).

### Cohort 2-Tissue microarray

#### Colorectal cancer tissue microarray construction

A tissue microarray of 221 unselected, non-consecutive colorectal cancer patients treated at the Second Department of Pathology, University of Athens between the years 2004 and 2006 was constructed at the Institute for Pathology, University Hospital of Basel. The use of this material was approved by the local ethics committee of the University of Athens.

Each patient had multiple tissue punches taken from formalin-fixed, paraffin-embedded blocks using a tissue cylinder with a diameter of 0.6 mm, which were subsequently transferred into one recipient paraffin block (3 × 2.5 cm^2^) using a homemade semi-automated tissue arrayer. Tissues were obtained from the tumour centre, the invasive tumour front within the representative area of most intense tumour budding in all sections of the tumour, as determined from corresponding H&E slides, the normal adjacent mucosa (if available) and the transitional zone where tumour and normal adjacent mucosa first interact (if available). Each patient on average had 5.1 tissue punches included on this array. The final tissue microarray contained 1079 tissues, namely 437 tissues from the tumour centre, 430 from the invasive front, 90 from normal adjacent mucosa and 122 from the transitional zone. For the purposes of this study, only tissue punches from the invasive tumour front per patient were analysed.

#### Clinico-pathological features

H&E slides were reviewed and histomorphological data included histological subtype, pT stage, pN stage, pM stage, tumour grade, and vascular and lymphatic invasion. Clinical data were retrieved from patient records and included age at diagnosis, gender, tumour location and follow up. Information on adjuvant therapy was available for all patients. Patients with TNM stage IV disease were removed from this study, thus 191 patients with stage I–III disease constituted the final patient cohort. Clinical outcome of interest was disease-specific survival time, which was available for all 191 patients. Median follow-up time is 35 months and 44 patients died of the disease. Patient characteristics are listed in Table 2.

#### Immunohistochemistry

Immunohistochemistry was carried out using anti-CD8 antibody at the Second Department of Pathology, University of Athens. Briefly, 5-*μ*m TMA sections were deparaffinised, and pre-treated with the Envision Flex Target Retrieval Solution pH8.8 (DM812, Dako) at 800°C for 6 min followed by incubation at room temperature for 10 min. After wash steps, peroxidase blocking was carried out for 10 min. Sections were again washed and then incubated with primary antibody against CD8 (DakoCytomation; clone CD8/144B) for 60 min. Subsequently, sections were washed with TBS and incubated with Real Envision Solution (Dako) for 30 min, then washed again in TBS and incubated with DAB-Chromogen for 10 min. After washing, sections were counterstained with haematoxylin for 2 min. Only peritumoural CD8+ T-lymphocytes in punches taken from the invasive tumour front were counted.

#### Cut-off score for low vs high budding, CD8+ and CD8+/ buds indices

All cut-off scores to classify patients as having a ‘low’ or ‘high’ index were obtained by receiver operating characteristic curve (ROC) analysis (Zlobec *et al*, 2007b). 50% of the data was randomly selected for this purpose. Using this method, a plot of the sensitivity and false positive rate (1-specificity) for discriminating between survivors and non-survivors was assessed. The most discriminating cut-off score was determined as the point on the ROC curve with the shortest distance to the coordinate (0, 1), namely with the maximum sensitivity and specificity for survival. The reliability of all cut-off scores was tested by re-sampling of the data (500 bootstrapped replications). The discriminatory ability of each feature for survival was also evaluated by analyzing the area under the ROC curve (AUC) and 95% confidence interval (CI).

#### Additional statistical analyses

The association of indices with categorical clinico-pathological features was obtained using the *χ*^2^ and Fisher's exact tests, where appropriate. Univariate survival analysis was carried out using the Kaplan–Meier and log-rank tests. Multivariable analysis was carried out using Cox's proportional hazards regression analysis after the verification of proportional hazards assumption. Hazard ratio (HR) and 95%CI were obtained to determine the prognostic effect of each index after adjustment. With logistic regression analysis, the odds ratios (OR) and 95%CI were obtained to determine the odds of death at 5 years with the use of different indices. All statistical analyses were carried out using SAS (V9, The SAS Institute, NC, Cary, USA).

## Results

### Cohort 1-Whole tissue sections

#### Tumour-budding index

**Figure 2:**
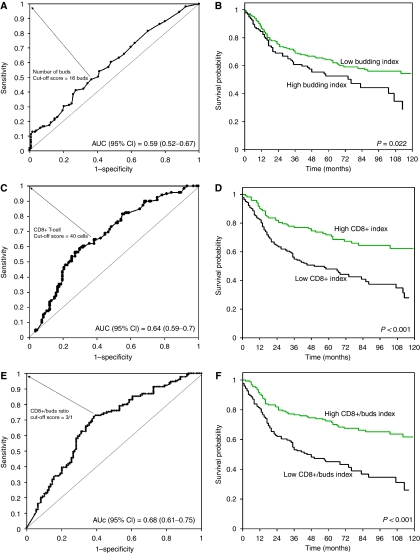
Cohort 1. (**A**, **C** and **E**): Receiver operating characteristic (ROC) curves highlighting the ability of tumour budding (**A**), CD8+ lymphocytes (**C**) and CD8+/ buds index (**E**) to discriminate survivors and non-survivors. AUC=area under the ROC curve. Larger the AUC, more discriminating the feature. Arrows are drawn from the point on the curve used to classify patients into ‘low’ or ‘high’ indices based on their proximity to the coordinate (0,1). (**B**, **D** and **F**) corresponding Kaplan–Meier survival curves for ‘low’ and ‘high’ indices.

A high tumour-budding index was defined, using ROC curve analysis, as at least 16 buds per × 40 field (Figure 2A). However, the ROC curve also highlights a relatively poor discrimination of survivors and non-survivors at 5-year follow up with an AUC value of 0.59 (95%CI: 0.52–0.67). A high-budding index was significantly associated with a more advanced pT stage (*P*=0.017), with the presence of lymph node metastasis (*P*=0.002), with the presence of vascular invasion (*P*=0.028) and with an infiltrating tumour border configuration (*P*=0.005) (Table 3). Patients with MSI-H cancers were significantly less prone to a high tumour-budding index (*P*=0.022), whereas no association was observed with *KRAS* or *BRAF* mutation. The odds of death from disease at 5 years in the group with a high-budding index was OR (95%CI)=1.89 (1.1–3.3) compared with those with a low index (*P*=0.022) (Table 4). In all, 5-year disease-specific survival rate of patients with high and low tumour-budding index was 53.0 (95%CI 42–63) and 63.9 (95%CI 57–70), suggesting a significantly worse prognosis in patients with high tumour-budding index (*P*=0.033) (Figure 2B). In patients with MSS/MSI-L tumours, a high-budding index was related to more advanced pT stage (*P*=0.019), pN stage (*P*=0.002), vascular invasion (*P*=0.004) and an infiltrating tumour border configuration (*P*=0.021) and worse survival in univariate analysis (*P*=0.02). In MSI-H patients, the high tumour-budding index was only related to an infiltrating tumour border configuration (*P*=0.015).

#### CD8+ index

A high CD8+ index was characterised by the presence of at least 40 CD8+ cells per × 40 field (Figure 2C). The discriminatory ability of CD8+ as indicated by the AUC was 0.64 (95%CI 0.59–0.7), suggesting an improvement compared with the budding index alone. The odds of death from disease at 5 years was OR (95%CI)=3.18 (1.8–5.5) in patients with a low compared with a high CD8+ index. A high CD8+ index was associated with significantly more frequent peritumoural lymphocytic inflammation at the tumour front (*P*=0.002), with MSI-H status (*P*=0.033) and marginally with *BRAF* mutation. Patients with a high CD8+ index demonstrated a significantly prolonged survival time compared with those with a low CD8+ index (*P*<0.001) (Figure 2D). In MSS/MSI-L and MSI-H patients, the CD8+ index was not linked to any features of tumour progression although in MSS/MSI-L those with a high CD8+ index experienced a significant prolonged survival time (*P*=0.011).

#### CD8+/ buds index

A ratio of at least three times more CD8+ T-cells *vs* the number of tumour buds led to a strong discrimination of survivors and non-survivors of colorectal cancer, as indicated by an AUC value of 0.68 (95%CI 0.61–0. 75) (Figure 2E). The odds of death at 5 years in patients with a low CD8+/ buds index compared with those with a high index was OR (95%CI)=4.12 (2.4–7.1) indicating that on average the odds of death was 4.12 times greater in patients with a low compared with a high CD8+/ buds index (Table 4). Patients with a high CD8+/ buds index demonstrated more favourable features, such as early T stage (*P*<0.001), absence of vascular invasion (*P*=0.009), presence of a pushing/expanding tumour border configuration (*P*=0.005), presence of peritumoural lymphocytic inflammation at the invasive front (*P*=0.007) and a marginal association with MSI-H status (*P*=0.052). Most notably, a considerable difference in survival time between patients with low and high CD8+/ buds index was observed with patients with a low index demonstrating a highly adverse prognosis (*P*<0.001) (Figure 2F). This prognostic difference was also maintained in the group of MSS/MSI-L patients (*P*=0.001).

#### Independent prognostic effects of budding, CD8+ and CD8+/ buds indices

A comparison of the performance of each of these indices in multivariable survival analysis is outlined in Table 5. The effect of each of these factors was re-evaluated along with age, gender, pT stage, pN stage, tumour grade, vascular invasion and the tumour border configuration. Analysis was thus carried out only for patients with complete clinico-pathological information for all these features (*n*=226, 101 deaths). The budding index was not found to add independent prognostic information when analysed along with these additional features (*P*=0.85), whereas the CD8+ index (*P*<0.001) and the CD8+/ buds index (*P*<0.001) were highly relevant as prognostic factors in multivariable analysis, with similar relative risks of death suggesting a comparable performance of these two features as prognostic indices.

In order to prevent over-fitting of multivariable models, only the most relevant prognostic factors were included into sub-group analyses of lymph node-negative patients (*n*=140, 42 deaths), as well as of those with colon (*n*=176; 79 deaths) or rectal cancers (*n*=94; 44 deaths). In lymph node-negative patients, multivariable analysis with pT stage, vascular invasion and tumour border configuration revealed independent prognostic effects for both the CD8+ index (*P*=0.001) and the CD8+/ buds index (*P*=0.002), but not for the budding index. Moreover, in patients with colon or rectal cancer, the CD8+ index (*P*=0.015 and *P*=0.006, respectively) as well as the CD8+/ buds index (*P*=0.002 and *P*=0.016, respectively) maintained a significant independent prognostic effect after adjusting for pT stage, pN stage and vascular invasion.

### Cohort 2-Tissue microarray

In Cohort 2, a high tumour-budding index in stages I–III patients was defined as at least six buds per punch and was related to a more advanced pT stage (*P*=0.029), more poorly differentiated tumours (*P*=0.003), presence of vascular invasion (*P*=0.045) and was highly related to a worse prognosis (*P*=0.003) (Table 6). The threshold value for a high CD8+ index was 13 cells per punch and was linked to lymph node negativity (*P*=0.053), early tumour grade (*P*=0.014), absence of vascular invasion (*P*=0.002) and a prolonged survival time compared with patients with a low CD8+ index (*P*=0.031).

**Figure 3:**
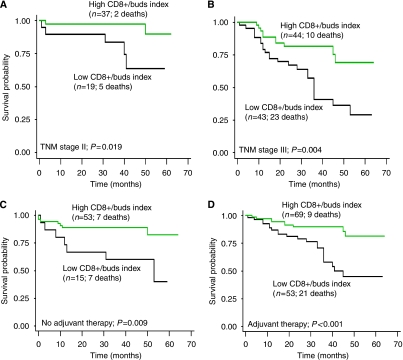
Cohort 2. Kaplan–Meier survival curves illustrating the poorer outcome in patients with a low CD8+/ buds index in (**A**) TNM stage II patients, (**B**) TNM stage III patients, (**C**) patients not receiving adjuvant therapy and (**D**) patients treated with adjuvant therapy.

A ratio of three times more CD8+ lymphocytes to the number of tumour buds was identified as the most discriminating cut-off value to classify survivors and non-survivors. Patients with a high CD8+/ buds index had tumours that were more early pT stage (*P*=0.001), lymph node-negative (*P*=0.055), early tumour grade (*P*<0.001) and vascular invasion negative (*P*=0.002). Low CD8+/ buds index, led to a highly unfavourable prognosis compared with patients with a high CD8+/ buds index (*P*<0.001). This difference was also maintained in sub-group analysis of TNM stage II (*P*=0.019) and stage III (*P*=0.004) patients (Figure 3A and B). In particular, in both untreated and treated patients, a low CD8+/ buds index was linked to a shorter survival time (*P*=0.009 and *P*<0.001, respectively) (Figure 3B and C). Multivariable analysis for the budding index, CD8+ index and CD8+/ buds index was carried out accounting for pN stage, vascular and lymphatic invasion and treatment effect. Results outlined in Table 7 highlight once more the independent adverse prognostic effect of a low CD8+/ buds index after adjusting for these additional factors.

## Discussion

The aim of this study was to investigate an alternative approach to reflect the dynamic at the tumour front of colorectal cancer using an index including tumour budding and CD8+ lymphocytes.

Briefly summarised, our results confirm the prognostic impact of the selected tumour and host-related factors. In both patient cohorts, a high tumour budding and a low CD8+ lymphocytes index were associated with tumour progression and worse survival. Indeed, tumour budding has previously been found to be associated with higher N stage, higher tumour grade and presence of vascular invasion, local tumour recurrence, distant metastasis as well as worse survival (Ueno *et al*, 2004a,2004b; Nakamura *et al*, 2005; Kazama *et al*, 2006; Prall, 2007; Ishikawa *et al*, 2008; Wang *et al*, 2009). There is also strong evidence for an association between a high number of CD8+ lymphocytes and absence of lymph node metastases, lower tumour grade, presence of peritumoural lymphocytes, mismatch repair-deficient status and better survival (Ropponen *et al*, 1997; Naito *et al*, 1998; Chiba *et al*, 2004; Galon *et al*, 2006; Koch *et al*, 2006; Baker *et al*, 2007).

The question, therefore, arises as to whether a CD8+ lymphocytes : budding index is more advantageous over the use of one out of the two parameters alone. With the exception of 5-year survival, a certain intra- and inter-group variability is observed when analysing the relationship of tumour budding or CD8+ lymphocytes with clinico-pathological parameters. In contrast, the use of a CD8+ lymphocytes : budding index better summarises these associations with key endpoints of tumour progression, namely presence of lymph node metastases, vascular invasion and worse survival, and moreover, in both cohorts. In addition, the CD8+/ buds index shows an increased discriminatory ability for identifying survivors and non-survivors at 5-year follow up compared with tumour budding or CD8+ lymphocytes count themselves. More specifically, patients with a low CD8+ lymphocyte : buds index have more than four-fold odds of death at 5 years compared with a three-fold or under two-fold risk for patients with low CD8+ counts or high number of tumour buds, respectively. In addition, the CD8+/ buds index maintained its significant impact on outcome in patients with stage II disease, in both colon and rectal cancer patients and also, independently of adjuvant therapy. Our previous work describes the absence of tumour budding in cases with marked peritumoural inflammatory reaction at the invasive margin of colorectal cancers and a strong correlation between this inflammation and the presence of TILs(Zlobec *et al*, 2007a). We hypothesised that an immune response to colorectal cancer could account for an improved prognosis in patients with abundant TIL count by specifically targeting budding cells, a so-called ‘nipping in the bud’ effect leading to improved prognosis. Although we cannot directly allude to the functional interaction of CD8+ lymphocytes and tumour budding in this study, our data nonetheless point to a strong link between favourable prognosis and a high CD8+ lymphocyte : tumour-budding index.

Several points in this study should still be considered. Although the impact of tumour budding on clinical outcome is validated by several groups, a standardised scoring system for this feature has not yet been established, although several similar methods have been proposed (Ueno *et al*, 2002; Shinto *et al*, 2005; Prall and Ostwald, 2007). This study highlights a new approach, which may be helpful in furthering the understanding of colorectal pathogenesis and prognosis, but requires validation by other research groups both in retrospective and prospective settings. Finally, our findings, particularly with regard to the test group may be somewhat influenced by the lack of clinical information regarding recurrence, metastasis and treatment although the majority of clinico-pathological features are adequately covered.

On the other hand, several factors strongly support the validity of our findings. The selection of parameters included in the proposed index is evidence based, as both tumour budding and the CD8+ lymphocytes have been tested and validated in colorectal cancer by different research groups. The index, which encompasses a tumour- and host-related feature, and therefore represents the more advantageous ‘multi-marker’ approach to prognosis, better reflects the tumour dynamic at the invasive front, compared with either feature alone. This study also benefits from the inclusion of two completely independent cohorts of colorectal cancer patients treated in Switzerland and Greece, respectively. In addition to whole tissue sections, we used the tissue microarray technique, a powerful tool for the investigation of novel or potential biomarkers, which has allowed us to evaluate hundreds of tissue cores for CD8+. Moreover, the evaluation of multiple tissue punches per tumour in this study exceeds the number of cores required for adequate representativity of tumour heterogeneity (Goethals *et al*, 2006). Although double staining was carried out for Cohort 1, only immunohistochemistry for CD8 was carried out on Cohort 2. These differences in methodology were chosen based on the ease of identification of buds. In whole tissue sections, budding can often be missed because of peritumoural inflammation, whereas tissue microarrays may aid the observer to focus on one specific area containing tumour buds. It is important to note that the independent prognostic effects of the CD8+/ buds index were found in both sub-groups despite the different laboratory circumstances and methodologies, thereby underlining the biological consistency of the CD8+/ buds index in colorectal cancer. Although we recognise that this particular index is unlikely to be a final solution for daily diagnostic practice, it may however serve as a basis that can be further extended to include other strong and validated ‘pro-tumour’ or ‘anti-tumour’. This approach could potentially lead to a new colorectal cancer prognostic score, which can be applied in addition to TNM staging, as is the case for the BRE score for breast cancers.

In conclusion, the CD8+ lymphocyte to tumour-budding index is an independent prognostic factor in colorectal cancer and represents biologically a ‘pro-/anti-tumour’ model that could be a promising approach for a future prognostic score in colorectal cancer.

## Figures and Tables

**Table 1:** Characteristics of patients in Cohort 1 (*n*=279)

**Clinico-pathological features**	**Frequency *N* (%)**
*Age (years) (n*=*279)*	
Mean, range	67.7, 37–96
	
*Tumour diameter (mm) (n=279)*	
Mean, range	54.0, 13–170
	
*Gender (n=279)*	
Male	122 (43.7)
Female	157 (56.3)
	
*Tumour location (n=279)*	
Left-sided	71 (25.4)
Right-sided	111 (39.8)
Rectum	97 (34.7)
	
*Histological subtype (n=277)*	
Adenocarcinoma	252 (91.0)
Mucinous	21 (7.6)
Other	4 (1.4)
	
*pT stage (n=274)*	
pT1	6 (2.2)
pT2	35 (12.8)
pT3	187 (68.3)
pT4	46 (16.8)
	
*pN stage (n=273)*	
pN0	140 (51.3)
pN1	76 (27.8)
pN2	57 (20.9)
	
*Tumour grade (n=274)*	
G1	5 (1.8)
G2	247 (90.2)
G3	22 (8.0)
	
*Vascular invasion (n=274)*	
Absent	206 (75.2)
Present	68 (24.8)
	
*Tumour border configuration (n=271)*	
Infiltrating	186 (68.6)
Pushing	85 (31.4)
	
*Peritumoural lymphocytic infiltration (n=274)*	
Absent	204 (74.5)
Present	70 (25.6)
	
*Microsatellite instability status (n=125)*	
Stable/low	95 (76.0)
Instable/high	30 (24.0)
	
*KRAS gene status (n=117)*	
Wild-type	82 (70.1)
Mutation	35 (29.9)
	
*BRAF gene status (n=106)*	
Wild-type	85 (80.2)
Mutation	21 (19.8)
	
*5-year survival (%) (n=279)*	
Rate (95%CI)	60.3 (54–66)

Continuous variables age and tumour diameter are described as the mean and range of values, whereas categorical variables are represented by the number of cases (% of cases). Survival time was obtained using the Kaplan–Meier method.

**Table 2:** Characteristics of patients in Cohort 2 (*n*=191)

	**Frequency *N* (%)**
*Age (years) (n=184)*	
Mean, range	67.9 (35–93)
	
*Tumour diameter (mm) (n=189)*	
Mean, range	4.6 (1.0–12.0)
	
*Gender (n=189)*	
Male	88 (46.6)
Female	101 (53.4)
	
*Tumour location (n=190)*	
Left-sided	113 (59.5)
Right-sided	27 (14.2)
Rectum	50 (26.3)
	
*Histological subtype (n=189)*	
Mucinous	24 (12.7)
Other	165 (87.3)
	
*pT stage (n=189)*	
pT1–2	52 (27.5)
pT3–4	137 (72.5)
	
*pN stage (n=189)*	
pN0	102 (54.0)
pN1–2	87 (46.0)
	
*TNM stage (n=189)*	
Stage I	45 (23.8)
Stage II	57 (30.2)
Stage III	87 (46.0)
	
*Tumour grade (n=189)*	
G1–2	122 (64.6)
G3	67 (35.5)
	
*Vascular or lymphatic invasion (n=191)*	
Absent	147 (91.1)
Present	17 (8.9)
	
*Adjuvant therapy (n=191)*	
None	69 (36.1)
Treated	122 (63.9)
	
*5-year survival (%) (n=191)*	
Rate (95%CI)	67.5 (57–76)

Continuous variables age and tumour diameter are described as the mean and range of values, whereas categorical variables are represented by the number of cases (% of cases). Survival time was obtained using the Kaplan–Meier method.

**Table 3:** Association of budding index, CD8+ index and CD8+/ buds index with clinico-pathological features – Cohort 1 (*n*=279)

	**Budding index**			**CD8+ index**			**CD8+/buds index**		
**Clinico-pathological feature**	**Low**	**High**	***P*-value**	**Low**	**High**	***P*-value**	**Low**	**High**	***P*-value**
*Gender*									
Female	107 (56.9)	50 (55.0)	0.797	61 (54.0)	87 (57.2)	0.598	72 (54.1)	85 (58.2)	0.492
Male	81 (43.1)	41 (45.1)		52 (46.0)	65 (42.8)		61 (45.9)	61 (41.8)	
									
*Tumour location*									
Left-sided	48 (25.5)	23 (25.3)	0.06	30 (26.5)	37 (40.7)	0.551	34 (25.6)	37 (25.3)	0.053
Right-sided	82 (43.6)	29 (31.9)		42 (37.2)	62 (40.8)		45 (33.8)	66 (45.2)	
Rectum	58 (30.9)	39 (42.9)		41 (36.3)	53 (34.9)		54 (40.6)	43 (29.5)	
									
*pT stage*									
pT1–2	34 (18.6)	7 (7.7)	0.017	15 (13.5)	20 (13.4)	0.983	15 (11.4)	26 (18.3)	0.107
pT3–4	149 (81.4)	84 (92.3)		96 (86.5)	129 (86.6)		117 (88.6)	116 (81.7)	
									
*pN stage*									
pN0	106 (57.9)	34 (37.8)	0.002	50 (45.5)	81 (54.4)	0.156	53 (40.5)	87 (61.3)	< 0.001
pN1–2	77 (42.1)	56 (62.2)		60 (54.6)	68 (45.6)		78 (59.5)	55 (38.7)	
									
*Tumour grade*									
G1–2	166 (90.7)	86 (94.5)	0.276	104 (63.7)	135 (90.6)	0.366	125 (94.7)	127 (89.4)	0.109
G3	17 (9.3)	5 (5.5)		7 (6.3)	14 (9.4)		7 (5.3)	15 (10.6)	
									
*Vascular invasion*									
Absent	145 (79.2)	61 (67.0)	0.028	79 (71.2)	115 (77.2)	0.271	90 (68.2)	116 (81.7)	0.009
Present	38 (20.8)	30 (33.0)		32 28.8)	34 (22.8)		42 (31.8)	26 (18.3)	
									
*Tumour border configuration*									
Infiltrating	114 (63.0)	72 (80.0)	0.005	80 (73.4)	102 (68.9)	0.435	100 (76.9)	86 (61.0)	0.005
Pushing	67 (37.0)	18 (20.0)		29 (26.6)	46 (31.1)		30 (23.1)	55 (39.0)	
									
*Peritumoural lymphocytic inflammation*									
Absent	137 (74.9)	67 (73.6)	0.825	93 (83.8)	100 (67.1)	0.002	108 (81.8)	96 (67.6)	0.007
Present	46 (25.1)	24 (26.4)		18 (16.2)	49 (32.9)		24 (18.2)	46 (32.4)	
									
*KRAS gene status*									
Wild-type	55 (75.3)	27 (61.4)	0.11	31 (64.6)	47 (74.6)	0.253	38 (64.4)	44 (75.9)	0.176
Mutation	18 (24.7)	17 (38.6)		17 (65.4)	16 (25.4)		21 (35.6)	14 (24.1)	
									
*BRAF gene status*									
Wild-type	54 (77.1)	31 (86.1)	0.273	36 (90.0)	45 (75.0)	0.063	41 (85.4)	44 (75.9)	0.219
Mutation	16 (22.9)	5 (13.9)		4 (10.0)	15 (25.0)		7 (14.6)	14 (24.1)	
									
*Microsatellite instability status (MSS)*									
MSS/MSI-L	54 (69.2)	41 (87.2)	0.022	43 (86.0)	47 (69.1)	0.033	51 (83.6)	44 (68.8)	0.052
MSI-H	24 (30.8)	6 (12.8)		7 (14.0)	21 (30.9)		10 (16.4)	20 (31.2)	
									
*Survival rate (%) (95% CI)*									
5-year	63.9 (57–70)	53.0 (42–63)	0.033	47.2 (38–56)	67.9 (60–75)	< 0.001	46.6 (38–55)	72.8 (65–79)	< 0.001

**Table 4:** Comparison of the discriminatory ability of each feature for identifying survivors and non-survivors at 5-year follow up

**Feature**	**Sensitivity**	**Specificity**	**Overall accuracy (95% CI)**	**OR (95% CI)**	***P*-value**
Budding count (high *vs* low)	0.413	0.737	0.59 (0.52–0.67)	1.89 (1.1–3.3)	0.022
CD8+ count (low *vs* high)	0.597	0.683	0.64 (0.59–0.7)	3.18 (1.8–5.5)	<0.001
CD8 : buds index (low *vs* high)	0.721	0.61	0.68 (0.61–0.75)	4.12 (2.4–7.1)	<0.001

Odds ratio (OR) indicate that the odds of disease-specific death at 5-years is 1.89 times higher in the high-budding count group compared with the low group; for CD8+ count, 3.18 times higher in the low compared with the high CD8+ count group and for the CD8 : buds index, is 4.12 times higher in those with a low compared with a high index.

**Table 5:** Comparison of relative risks of buds, CD8+ and CD8+/ buds index in multivariable analysis with age, gender, pT stage, pN stage, tumour grade, vascular invasion and tumour border configuration in Cohort 1

	**Buds index only**		**CD8+ index only**		**CD8+/ buds index**	
	**HR (95% CI)**	***P*-value**	**HR (95% CI)**	***P*-value**	**HR (95% CI)**	***P*-value**
*Index*						
Low	1.0	0.85	1.0	<0.001	1.0	<0.001
High	1.04 (0.7–1.6)		0.45 (0.3–0.7)		0.44 (0.3–0.7)	
						
*Age (years)*						
Baseline	1.0	0.041	1.0	0.026	1.0	0.021
Increasing by year	1.02 (1.0–1.1)		1.02 (1.0–1.1)		1.02 (1.0–1.1)	
						
*Gender*						
Male	1.0	0.468	1.0	0.507	1.0	0.443
Female	0.86 (0.6–1.3)		0.87 (0.6–1.3)		0.86 (0.6–1.3)	
						
*pT stage*						
pT1–2	1.0	0.042	1.0	0.048	1.0	0.022
pT3–4	2.41 (1.0–5.6)		2.35 (1.0–5.5)		2.69 (1.2–6.3)	
						
*pN stage*						
pN0	1.0	<0.001	1.0	<0.001	1.0	0.001
pN1–2	2.27 (1.5–3.5)		2.12 (1.4–3.3)		2.06 (1.4–3.2)	
						
*Tumour grade*						
G1–2	1.0	0.205	1.0	0.738	1.0	0.873
G3	0.63 (0.3–1.3)		0.88 (0.4–1.9)		0.94 (0.4–2.0)	
						
*Vascular invasion*						
Absent	1.0	<0.001	1.0	0.001	1.0	0.014
Present	2.2 (1.4–3.5)		2.1 (1.3–3.3)		1.79 (1.1–2.8)	
						
*Tumour border configuration*						
Pushing	1.0	0.758	1.0	0.945	1.0	0.915
Infiltrating	1.09 (0.6–1.8)		1.02 (0.6–1.8)		1.03 (0.6–1.8)	

**Table 6:** Association of budding index, CD8+ index and CD8+/ buds index with clinico-pathological features – Cohort 2 (*n*=191)

	**Budding index**			**CD8+ index**			**CD8+/ buds index**		
**Clinico-pathological feature**	**Low (*n*=131)**	**High (*n*=60)**	***P*-value**	**Low (*n*=69)**	**High (*n*=121)**	***P*-value**	**Low (*n*=68)**	**High (*n*=122)**	***P*-value**
*Gender*									
Male	65 (50.4)	23 (38.3)	0.122	34 (49.3)	54 (54.4)	0.606	33 (48.5)	55 (45.8)	0.722
Female	64 (49.6)	37 (61.7)		35 (50.7)	65 (54.6)		35 (51.5)	65 (54.2)	
									
*Tumour location*									
Left-sided	80 (61.5)	33 (55.0)	0.311	38 (55.1)	74 (61.7)	0.673	35 (51.5)	77 (63.6)	0.054
Right-sided	20 (15.4)	7 (11.7)		11 (15.9)	16 (13.3)		8 (11.8)	19 (15.7)	
Rectum	30 (23.1)	20 (33.3)		20 (16.0)	30 (25.0)		25 (36.8)	25 (20.7)	
									
*pT stage*									
pT1–2	42 (32.3)	10 (17.0)	0.029	15 (21.7)	37 (31.1)	0.167	9 (13.4)	43 (35.5)	0.001
pT3–4	88 (67.7)	49 (83.0)		54 (78.3)	82 (68.9)		58 (86.6)	78 (64.5)	
									
*pN stage*									
pN0	79 (71.2)	23 (67.7)	0.694	30 (60.0)	71 (75.5)	0.053	24 (58.5)	77 (74.8)	0.055
pN1–2	32 (28.8)	11 (32.4)		20 (40.0)	23 (24.5)		17 (41.5)	26 (25.2)	
									
*Tumour grade*									
G1–2	93 (71.5)	29 (49.2)	0.003	37 (53.6)	85 (71.4)	0.014	29 (43.3)	93 (76.9)	<0.001
G3	37 (28.5)	30 (50.8)		32 (46.4)	34 (28.6)		38 (56.7)	28 (23.1)	
									
*Vascular invasion*									
Present	8 (6.1)	9 (15.0)	0.045	12 (17.4)	5 (4.1)	0.002	12 (17.7)	5 (4.1)	0.002
Absent	123 (93.9)	51 (85.0)		57 (82.6)	116 (95.9)		56 (82.4)	117 (95.9)	
									
*Survival rate (%) (95%CI)*									
5 years	75.0 (62–84)	52.9 (36–67)	0.003	49.4 (30–66)	77.1 (66–85)	0.031	43.8 (26–60)	81.5 (69–89)	<0.001

**Table 7:** Comparison of relative risks of buds, CD8+ and CD8+/ buds index in multivariable analysis with pN stage, vascular and lymphatic invasion and adjuvant therapy in Cohort 2

	**Buds index only**		**CD8+ index only**		**CD8+/ buds index**	
**Cohort 2**	**HR (95% CI)**	***P*-value**	**HR (95% CI)**	***P*-value**	**HR (95% CI)**	***P*-value**
*Index*						
Low	1.0	0.029	1.0	0.473	1.0	0.005
High	1.96 (1.1–3.6)		0.79 (0.4–1.5)		0.39 (0.2–0.8)	
						
*pN stage*						
pN0	1.0	<0.001	1.0	<0.001	1.0	0.002
pN1–2	4.11 (1.8–9.2)		4.41 (2.0–9.7)		3.58 (1.6–7.9)	
						
*Vascular/lymphatic invasion*						
Absent	1.0	<0.001	1.0	0.006	1.0	0.007
Present	3.41 (1.7–7.1)		2.97 (1.4–6.4)		2.76 (1.3–5.7)	
						
*Adjuvant therapy*						
Untreated	1.0	0.057	1.0	0.068	1.0	0.076
Treated	0.5 (0.2–1.0)		0.52 (0.3–1.1)		0.53 (0.3–1.1)	
